# β-elemene inhibited expression of DNA methyltransferase 1 through activation of ERK1/2 and AMPKα signalling pathways in human lung cancer cells: the role of Sp1

**DOI:** 10.1111/jcmm.12476

**Published:** 2015-01-19

**Authors:** ShunYu Zhao, Jingjing Wu, Fang Zheng, Qing Tang, LiJun Yang, Liuning Li, WanYin Wu, Swei Sunny Hann

**Affiliations:** a Laboratory of Tumor Biology, The second Clinical Medical Collage, University of Guangzhou Traditional Chinese MedicineGuangdong Province, China; b Department of Medical Oncology, Guangdong Provincial Hospital of Chinese Medicine, The second Clinical Medical Collage, University of Guangzhou Traditional Chinese MedicineGuangzhou, Guangdong Province, China

**Keywords:** β-elemene, ERK1/2, AMPKα, Sp1, DNMT1, NSCLC

## Abstract

β-elemene, a compound derived from *Rhizoma zedoariae*, is a promising new plant-derived drug with broad-spectrum anticancer activity. However, the underlying mechanism by which this agent inhibits human lung cancer cell growth has not been well elucidated. In this study, we showed that β-elemene inhibits human non-small cell lung carcinoma (NSCLC) cell growth, and increased phosphorylation of ERK1/2, Akt and AMPKα. Moreover, β-elemene inhibited expression of DNA methyltransferase 1 (DNMT1), which was not observed in the presence of the specific inhibitors of ERK (PD98059) or AMPK (compound C). Overexpression of DNMT1 reversed the effect of β-elemene on cell growth. Interestingly, metformin not only reversed the effect of β-elemene on phosphorylation of Akt but also strengthened the β-elemene-reduced DNMT1. In addition, β-elemene suppressed Sp1 protein expression, which was eliminated by either ERK1/2 or AMPK inhibitor. Conversely, overexpression of Sp1 antagonized the effect of β-elemene on DNMT1 protein expression and cell growth. Taken together, our results show that β-elemene inhibits NSCLC cell growth *via*ERK1/2- and AMPKα-mediated inhibition of transcription factor Sp1, followed by reduction in DNMT1 protein expression. Metformin augments the effect of β-elemene by blockade of Akt signalling and additively inhibition of DNMT1 protein expression. The reciprocal ERK1/2 and AMPKα signalling pathways contribute to the overall responses of β-elemene. This study reveals a potential novel mechanism by which β-elemene inhibits growth of NSCLC cells.

## Introduction

Lung cancer especially non-small cell lung cancer (NSCLC) is the most common type of malignancy and the leading cause of cancer-related mortality worldwide with low 5-year survival rate 1. At the time of presentation, most patients are at an advanced stage with poor outcome. Traditional Chinese Medicine (TCM) plays an important role in protecting cancer patients against suffering from complications, helping patients to live well, assisting in supportive and palliative care by reducing side effects of conventional treatment and improving quality of life 2. However, the molecular mechanisms by which TCM in improving the therapeutic efficiency against the lung malignancies remains poorly understood.

β-elemene (1-methyl-1-vinyl-2,4-diisopropenyl-cyclohexane), a naturally occurring compound isolated from the TCM herb *Curcumae Radix*, has been used to target various solid tumours including lung cancer 3,4. β-elemene has been shown to inhibit the growth and DNA synthesis of multiple types of tumour cells, resulting in the apoptosis or suppressed growth of tumours without severe side effects 3,5. However, the underlying mechanisms associated with its efficacy in targeting lung cancer are largely unknown.

Tumour suppressor gene silencing by DNA hypermethylation contributes to tumourigenesis in various cancer types. This aberrant methylation may be because of increased expression and activity of methyltransferases, which catalyse the transfer of methyl groups from s-adenosylmethionine to cytosines in CpG dinucleotides. DNA methyltransferase 1 (DNMT1) is a major enzyme involved in the somatic inheritance of DNA methylation and thus plays a critical role in epigenomic stability. Dysregulation of DNMT1 is implicated in a variety of diseases 6, and aberrant methylation contributes to the pathogenesis of human cancers 7. In addition, increased expression of DNMT1 has been found in several tumour types including lung and caused silence of tumour suppressor genes 8–10. Thus, inhibition of DNMT1 may represent a promising approach for the prevention and treatment of many cancers 10–13.

In this study, we investigated the potential mechanism of β-elemene for its efficacy in suppressing lung cancer cell growth. We showed for the first time that β-elemene inhibits growth of NSCLC cells *via* both extracellular signal-regulated kinase 1/2 (ERK1/2)- and AMP-activated protein kinase alpha (AMPKα)-mediated inhibition of transcription factor Sp1, followed by reduction in DNMT1 expression.

## Materials and methods

### Reagents and cell cultures

Monoclonal antibodies specific for total ERK1/2, AMPKα, Akt and the phosphor-forms were purchased from Cell Signaling Technology Inc. (Beverly, MA, USA). The Sp1 and DNMT1 antibodies were obtained from Epitomics (Burlingame, CA, USA). PD98059 (ERK inhibitor), compound C (a special inhibitor of AMPK) and metformin (an activator of AMPK) were purchased from Merck Millipore (Darmstadt, Germany), MTT powder was purchased from Sigma-Aldrich (St. Louis, MO, USA). Sp1 small interfering RNAs (siRNAs) were obtained from Santa Cruz (Santa Cruz, CA, USA). Lipofectamine 3000 reagent was purchased from Invitrogen (Carlsbad, CA, USA). β-elemene was purchased from Chengdu Must Bio-technology Company (Chengdu, Sichuan, China). The drugs were freshly diluted to the final concentration with culture medium before treatment. Human lung adenocarcinoma cells (PC9, H1299, H1650, A549, H358 and H1975) and one bronchial epithelial cell line BEAS-2B were obtained from the Chinese Academy of Sciences Cell Bank of Type Culture Collection (Shanghai, China) and the Cell Line Bank at the Laboratory Animal Center of Sun Yat-sen University (Guangzhou, China). The cells were cultured at 37°C in a humidified atmosphere containing 5% CO_2_. The culture medium consisted of RPMI 1640 medium (Gibco, Beijing, China) supplemented with 10% (v/v) heat-inactivated foetal bovine serum (Thermo Fisher Scientific Inc, MA, USA), 100 μg/ml streptomycin and 100 U/ml penicillin. When cells reached 70% confluence, they were digested with 0.25% trypsin for passage for the following experiments.

### Cell viability assay

Cell viability was measured using the 3-(4, 5-dimethylthiazol-2-yl)-2, 5-diphenyltetrazolium bromide (MTT) assay. Briefly, NSCLC cells were harvested, counted and seeded into a 96-well microtitre plate, 2.5 × 10^3^ cells/well. The cells were treated with increasing concentrations of β-elemene for up to 72 hrs. After incubation, 20 μl MTT solution (5 g/l) was added to each well and NSCLC cells were incubated at 37°C for an additional 6 hrs. Supernatant was removed, then 150–200 μl solvent dimethyl sulfoxide was added to each well and oscillated for 10 min. Absorbance at 530 nm was determined through the use of ELISA reader (Perkin Elmer, Victor X5, Waltham, MA, USA). Cell viability (%) was calculated as (absorbance of test sample/absorbance of control) ×100%.

### Western blot analysis

The whole cell lysates were obtained from cells and protein concentrations were determined using the Bio-Rad (Hercules, CA, USA) protein assay. Afterwards, whole cell lysates were solubilized in 4× SDS sample buffer and separated on 10% SDS polyacrylamide gels. Membranes (Millipore, Billerica, MA, USA) were incubated with antibodies against ERK1/2, AMPKα, pERK1/2, p-AMPKα, Sp1 and DNMT1 (1:1000). The membranes were washed and incubated with a secondary antibody raised against rabbit IgG conjugated to horseradish peroxidase (Cell Signaling, Beijing, China). The membranes were washed again and transferred to freshly made ECL solution (Immobilon Western; Millipore, Shanghai, China), followed by observing signals under the Gel Imagine System (Bio-Rad) for up to 1 min., or exposed to X-ray film.

### Treatment with Sp1 small interfering RNAs (siRNAs)

For transfection, cells were seeded in six-well or 96-well culture plates in RPMI 1640 medium containing 5% FBS (no antibodies), grown to 60–70% confluence before incubation with siRNAs. Sp1 and control siRNAs (up to 25 nM) were transfected using the lipofectamine 3000 reagent according to the manufacturer's instructions and incubated with MEM medium for 30 min. at room temperature before the mixture was added to the cells. After culturing for up to 30 hrs, the cells were washed and resuspended in fresh media in the presence or absence of β-elemene for an additional 24 hrs for all other experiments.

### Electroporated transfection assays

NSCLC cells (5 × 10^7^ cells/ml) were transferred into conical tubes and centrifuged at 140 ×g for 10 min. After centrifuging, medium was removed and the cells were washed with 1× PBS, and centrifuged again at 1200 r.p.m. for 5 min. Afterwards, the PBS was aspirated and added Bio-Rad Gene Pulser electroporation buffer. After resuspending the cells, the desired control (pCMV-6) or DNMT1 expression vector (RG226414, pCMV6-AC-GFP, obtained from Rockville, Inc. MD, USA), control (pcDNA3.1) and Sp1 overexpression vector (pcDNA3.1Sp1/flu, kindly provided by Dr. Thomas E. Eling (NIEHS, Research Triangle Park, NC) 14 at a final concentration of 2 μg/ml were added and the electroporation plate were put in the MX cell plate chamber and closed the lid in Gene Pulser II Electroporation System (Bio-Rad). The electroporation conditions on the plates to deliver 160 V/5 msec. square wave were adjusted until reaching the optimum. After electroporation was completed, the cells were transferred to a culture plate. We typically transfer each 150–200 μl electroporation sample to a six-well tissue culture plate containing 2–3 ml RPMI 1640. Cells were incubated for 48 hrs at 37°C, then treated with β-elemene for an additional 24 hrs.

### Statistical analysis

All experiments were repeated at least three times. All data are expressed as mean ± SD. Differences between groups were assessed by one-way anova and significance of difference between particular treatment groups was analysed using Dunnett's multiple comparison tests (GraphPadPrism 5.0 software, LaJolla, CA, USA). Asterisks showed in the figures indicate significant differences in experimental groups in comparison with the corresponding control. *P* < 0.05 was considered statistically significant.

## Results

### β-elemene inhibits growth of human NSCLC cells in the time- and dose-dependent manner

**Fig 1:**
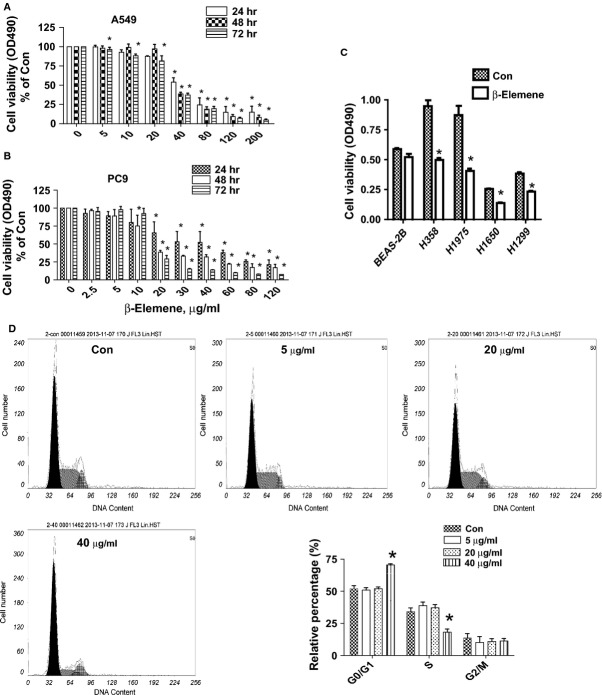
β-elemene inhibits human NSCLC cell growth in the time- and dose-dependent manner. A549 (A) and PC9 (B) cells were treated with increased concentrations of β-elemene for up to 72 hrs to examine the cell viability. (C) NSCLC cell lines indicated were treated with β-elemene (40 μg/ml) for 48 hrs. The cell viability was determined using the MTT assay as described in the Materials and Methods Section and was expressed as percentage of control in the mean ± SD of three separate experiments. *indicates significant difference as compared to the untreated control group (*P* < 0.05). (D) A549 cells were treated with increased doses of β-elemene for 24 hrs. Afterwards, the cells were collected and processed for analysis of cell cycle distribution by flow cytometry after propidium iodide (PI) staining. And the percentages of the cell population in each phase (G0/G1, S and G2/M) of cell cycle were assessed by Multicycle AV DNA Analysis Software. Data are expressed as a percentage of total cells. Values are given as the mean ± SD from three independent experiments performed in triplicate. *indicates significant difference as compared to the untreated control group (*P* < 0.05).

We first detected the effect of β-elemene on cell growth in NSCLC cells by MTT assay. As show in Figure1A and B, β-elemene decreased the cell viability in a dose- and time-dependent manner with maximal dose of 40 μg/ml observed at 48 hrs in A549 and PC9 cells. Similar results were also observed in other NSCLC cell lines (Fig.1C). To further examine the effects of β-elemene on cell proliferation, cell cycle phase distribution of NSCLC cells treated with increased doses of β-elemene for 24 hrs was analysed by flow cytometry after propidium iodide staining. We showed that, compared with the untreated control cells, β-elemene significantly increased the proportion of cells at G0/G1 phase, while the proportion of cells at S phases were reduced (Fig.1D) suggesting that β-elemene induced cell cycle arrest in G0/G1 phase in A549 cells.

### β-elemene increased phosphorylation of Akt, ERK1/2 and AMPKα in the time-dependent fashion

**Fig 2:**
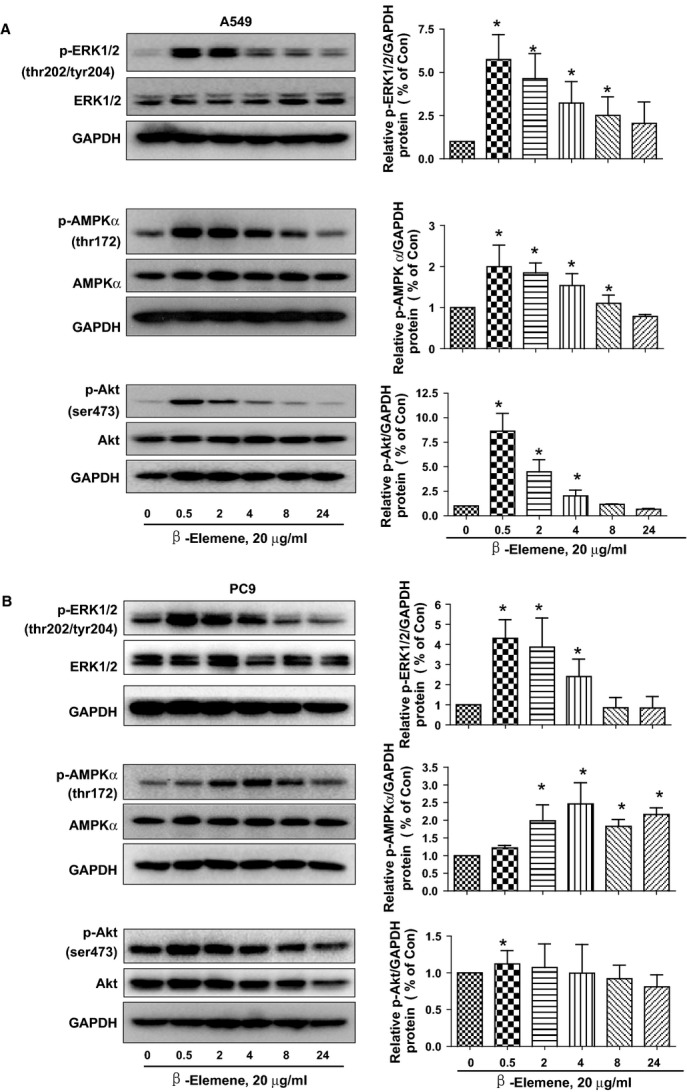
β-elemene increased phosphorylation of ERK1/2 and AMPKα in the time-dependent manner. A549 (A) and PC9 (B) cells were treated with β-elemene (20 μg/ml) in the indicated times, and cell lysate was harvested and the expression of the phosphorylated or total protein of ERK1/2 (upper), AMPKα (middle) and Akt (lower), were measured by Western blot analysis using corresponding antibodies. GAPDH was used as loading control. The bar graphs represented the densitometry results of p-ERK, p-Akt or AMPKα/GAPDH as mean ± SD of at least three separate experiments. *indicates significant difference from untreated control cells (*P* < 0.05).

We next tested the signalling pathways that mediated the effect of β-elemene on cell growth. Phosphatidylinositol 3-kinase (PI3-K)/Akt, ERK1/2 and AMPK signalling pathways have been shown to be involved in cell growth depending on the cell types and stimulus. We showed that β-elemene increased the phosphorylation of ERK1/2 and AMPKα, and unexpectedly, Akt in a time-dependent fashion in A549 and PC9 cells (Fig.2A and B). Note that the expression of total ERK1/2, AMPKα and Akt proteins had no significant changes (Fig.2A and B).

### β-elemene inhibited protein expression of DNMT1 in the does-dependent fashion; overexpression of DNMT1 reversed the effect of β-elemene on cell growth inhibition

**Fig 3:**
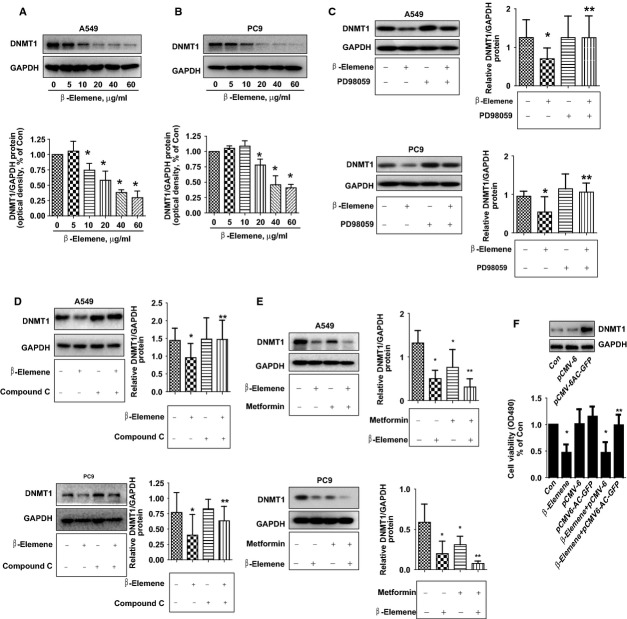
β-elemene inhibited protein expression of DNMT1 in the dose-dependent manner, overexpression of DNMT1 reversed the effect of β-elemene on cell growth. A549 (A) and PC9 (B) cells were exposed to increased concentrations of β-elemene for 24 hrs. Afterwards, the expression of DNMT1 protein were detected by Western blot. A549 and PC9 cells were treated with PD98059 (20 μM; C) or compound C (20 μM; D) for 2 hrs before exposure of the cells to β-elemene (20 μg/ml) for an additional up to 24 hrs. Afterwards, the expression of DNMT1 protein was detected by Western blot. (E) A549 and PC9 cells were treated with metformin (20 μM) and β-elemene (20 μg/ml) for up to 24 hrs. Afterwards, the expression of DNMT1 protein was detected by Western blot. The bar graphs represent the mean ± SD of DNMT1/GAPDH of three independent experiments. (F) Cells were transfected with control or DNMT1 expression vector for 24 hrs before exposing the cells to β-elemene for an additional 24 hrs. Afterwards, the expression of DNMT1 protein and cell growth were detected by Western blot and MTT assays, respectively. Values in bar graphs were given as the mean ± SD from three independent experiments performed in triplicate. * indicates significant difference as compared to the untreated control group (*P* < 0.05). ** indicates significant difference from β-elemene treated alone (*P* < 0.05).

β-elemene showed to inhibit cancer cell growth through distinct mechanisms in other reports 4,15,16. In this study, we found that β-elemene inhibited protein expression of DNMT1, a ubiquitous nuclear enzyme that plays an important role in epigenetically regulated gene expression, in the does-dependent fashion in both A549 and PC9 cells (Fig.3A and B). Interestingly, the specific inhibitors of ERK1/2 (PD98059) and AMPK (compound C) blocked β-elemene-reduced DNMT1 protein expression in A549 and PC9 cells (Fig.3C and D). Similar results were also observed by using siRNAs methods silencing of ERK1/2 and AMPKα (data not shown). Conversely, metformin, an activator of AMPK, further decreased DNMT1 protein expression in the presence of β-elemene in A549 and PC9 cells (Fig.3E). Moreover, A549 cells exogenous expression of DNMT1 showed to reverse the inhibitory effect of β-elemene on cell growth as determined by MTT assays (Fig.3F). Overall, the results above indicated that activation of ERK1/2 and AMPKα involved in the β-elemene-reduced DNMT1 protein and that expression of DNMT1 was required in the β-elemene-inhibited lung cancer cell growth.

### While PD98059 or compound C had little effect on influencing the effect of β-elemene on phosphorylation of AMPKα or ERK1/2, respectively; metformin reversed the effect of β-elemene on phosphorylation of Akt

**Fig 4:**
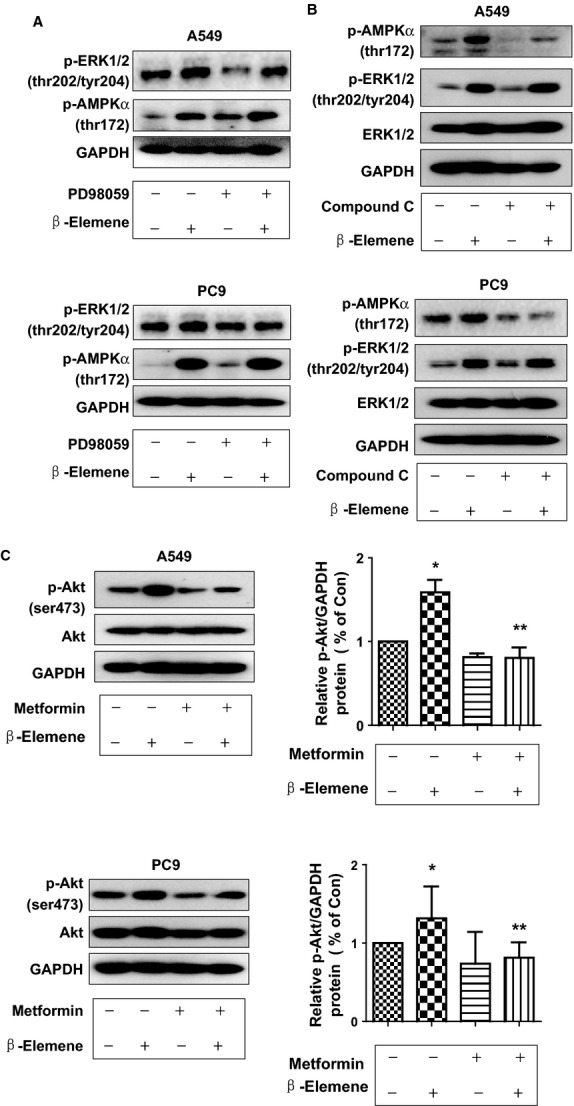
While PD98059 or compound C had little effect on influencing the effect of β-elemene in phosphorylation of AMPKα or ERK1/2, metformin reversed the effect of β-elemene on phosphorylation of Akt. A549 (A) and PC9 (B) cells were treated with PD98059 (20 μM) or compound C (20 μM) for 2 hrs before exposure of the cells to β-elemene (20 μg/ml) for an additional 24 hrs. Afterwards, the expression of p-ERK1/2 and p-AMPKα protein and their total forms was detected by Western blot. (C) A549 and PC9 cells were treated with metformin (5 mM) and β-elemene (20 μg/ml) for up to 2 hrs. Afterwards, the expression of p-Akt and p-AMPKα protein and total ones were detected by Western blot. The bar graphs represent the mean ± SD of p-Akt/GAPDH of three independent experiments. * indicates significant difference from untreated control cells. ** indicates significant difference from β-elemene treated alone (*P* < 0.05).

Interestingly, we found that, while PD98059 or compound C had little effect on influencing β-elemene-induced phosphorylation of AMPKα or ERK1/2, respectively (Fig.4A and B), metformin reversed the effect of β-elemene on phosphorylation of Akt in A549 and PC9 cells (Fig.4C). The findings indicated that a reciprocal signalling of ERK1/2 and AMPKα, and the inhibition of Akt by metformin may contribute to facilitate the overall responses of β-elemene.

### β-elemene inhibits transcription factor Sp1 protein expression, which was abolished by either ERK or AMPK inhibitors

**Fig 5:**
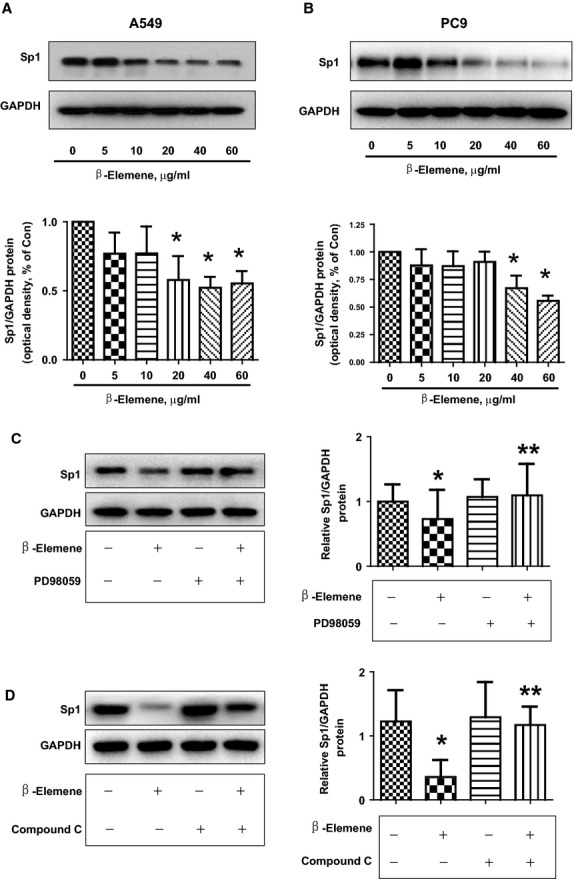
β-elemene inhibits transcription factor Sp1 protein expression, which was abrogated by the either ERK or AMPK inhibitor. A549 (A) and PC9 (B) cells were exposed to increased concentration of β-elemene for 24 hrs, followed by measuring the protein expression of Sp1 by Western blot. The bar graphs represent the mean ± SD of Sp1/GAPDH of three independent experiments. A549 cells were treated with PD98059 (20 μM; C) or compound C (20 μM; D) for 2 hrs before exposure of the cells to β-elemene (20 μg/ml) for an additional 24 hrs. Afterwards, the expression of Sp1 protein was detected by Western blot.

Previous studies reported that DNMT1 gene promoter contain transcription factor binding sites including Sp1, which regulated the expression and function of DNMT1 in several cell systems 17–19. This was for this reason we tested the role of Sp1. We showed that β-elemene inhibited Sp1 protein expression in a dose-dependent manner in A549 and PC9 cells (Fig.5A and B). Furthermore, the ERK1/2 and AMPK inhibitors abrogated the effect of β-elemene on Sp1 expression in A549 cells (Fig.5C and D). Together, the results suggested that activation of these kinases involved in regulation of Sp1 expression by β-elemene.

### While silencing of Sp1 showed no further effect, exogenous expression of Sp1 overcame the effect of β-elemene on DNMT1 expression and cell growth

**Fig 6:**
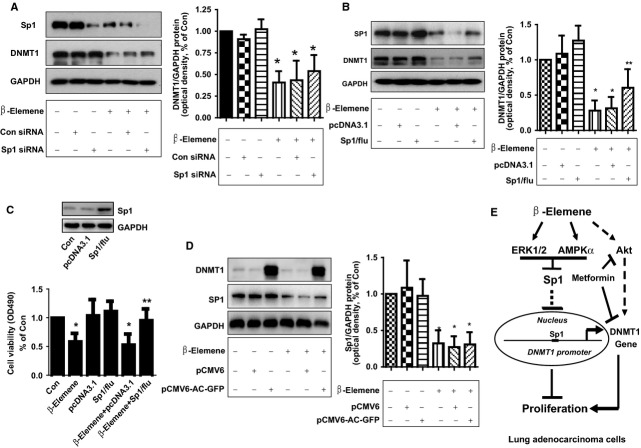
Overexpression of Sp1 reversed the effect of β-elemene on DNMT1 expression and cell growth, while silencing of Sp1 had no further effect. (A) A549 cells were transfected with control or Sp1 siRNAs (25 nM each) for 24 hrs prior to exposure of the cells to β-elemene for an additional 24 hrs. Afterwards, Western blot analysis was used measure the protein levels of Sp1 using corresponding antibodies. (B and C) A549 cells were transfected with control (pcDNA3.1) and Sp1 overexpression vector (pcDNA3.1Sp1/flu) for 24 hrs before exposing the cells to β-elemene for an additional 24 hrs. Afterwards, Sp1 protein expressions (B) and cell viability (C) were determined using Western blot and MTT assays. (D) A549 cells were transfected with control (pCMV6) and DNMT1 overexpression vector (pCMV6-AC-GFP) for 24 hrs before exposing the cells to β-elemene for an additional 24 hrs. Afterwards, Sp1 and DNMT1 protein expressions were determined using Western blot. Values in bar graphs were given as the mean ± SD from three independent experiments performed in triplicate. * indicates significant difference as compared to the untreated control group (*P* < 0.05). ** indicates significant difference from β-elemene treated alone (*P* < 0.01). (E) Diagram demonstrates that β-elemene inhibits NSCLC cell growth *via*ERK1/2- and AMPKα-mediated inhibition of transcription factor Sp1, followed by reduction in DNMT1 protein expression. The reciprocal signalling of ERK1/2 and AMPKα contributes to the overall responses of β-elemene. In addition, blockade of Akt signalling and concomitantly inhibition of DNMT1 by metformin augment the overall response of β-elemene.

In addition, we found that, while silencing of Sp1 had no further effect (Fig.6A), exogenous expression of Sp1 transfected into the cells resisted to the effect of β-elemene on DNMT1 expression (Fig.6B) and cell growth (Fig.6C). As expected, we also showed that overexpression of DNMT1 had no effect on β-elemene-reduced Sp1 protein expression (Fig.6D). Collectively, these results indicated that Sp1, acted as an upstream of DNMT1, played a critical role in mediating the overall responses of β-elemene in this process.

## Discussion

The major advantages of β-elemene as an anticancer drug have broad-spectrum anti-tumour effects in several types of cancer with fewer side effects 3,20,21. β-elemene emulsion injection as an adjunctive treatment for human cancers, including lung, has been reported in clinic studies, and the combination of chemotherapy with β-elemene treatment has been shown to improve quality of life and prolong survival of cancer patient 22,23. The dose ranges of β-elemene used in human lung cancer study were mostly from 400 to 800 mg/m^2^ in combination with chemotherapeutics, while the information for the serum and/or tissue concentration of β-elemene have not been observed 23. Nevertheless, the underlying mechanisms associated with its efficacy in inhibiting human lung cancer remain to be elucidated. In this study, we demonstrated that β-elemene inhibited NSCLC cell growth in the time- and dose-dependent manner, with an additional novel signalling pathway and potential molecular targets involved in this process (see below). The concentrations of β-elemene used here were consistent with or even lower than those reported by others demonstrating substantial growth inhibition of different types of cancer cells 21,24,25.

Multiple signalling pathways and potential target genes that mediated the overall response of β-elemene in inhibition of growth and induction of apoptosis of lung cancer cells have been reported 3,26. Consistent with this, our results demonstrated that activation of not only ERK1/2, but also, for the first time, AMPKα were involved in the effect of β-elemene on inhibition of Sp1 and DNMT1 protein expression. AMPK is the central component of protein kinase cascade that plays a key role in the regulation of energy control. Reported data showed that activated AMPK induced catabolic metabolism and suppressed the anabolic state, thereby inhibiting cell proliferation and potentially acting a tumour suppressor 27. Our current findings suggested that the reciprocal ERK1/2 and AMPKα signalling pathways contributed to the overall response of β-elemene in the control of lung cancer cell growth. The activation and interaction of these kinases signalling in mediating the physiopathological responses in regulation of cancer cell growth have been reported in other studies 28,29 demonstrating the critical roles of the complicated kinase networks in implication of gene expression and cancer cell survival. Of note, recent studies suggested that AMPK could exert dual roles in cancer biology depending on environment 30. We reasoned that the truly function of AMPK in suppressing tumour formation and growth needs to be determined.

Unexpectedly, we showed that β-elemene induced phosphorylation of Akt, a downstream effector of phosphatidylinositol 3-kinase (PI3-K), which was consistent with other report 26, suggesting that Akt signal was also involved in β-elemene-induced apoptosis in lung cancer cells 26. As opposite results also observed in other studies 31, we believed that more studies are required to better explore the role of the Akt signalling pathway in response to β-elemene. On the other hand, we demonstrated that metformin, which had anti-lung cancer properties 32, antagonized the effect of β-elemene on phosphorylation of Akt. This synergy might result in an augmented effect in the inhibition of lung cancer cell growth. Whether the AMPK-dependent signalling pathway was involved in effects of metformin on Akt required to be determined as AMPK-independent pathway was reported in other cell system 33,34. Also, additional experiments are warranted to further elucidate the combing response of these agents in control of human lung cancer cells.

Interestingly, our results demonstrated a central role of DNMT1 expression that mediated the effect of β-elemene on NSCLC cell growth suggesting the potential target of β-elemene and involvement of DNMT1 in lung cancer cell viability, which were never been reported before. Elevated expression of DNMT1 was found in various cancers including lung and antagonized the functions of tumour suppressor genes 8–10. Thus, re-expression of methylation silenced tumour suppressors *via* inhibition of DNMT1 has emerged as a potential therapeutic strategy against cancer. Reports demonstrated that targeting DNMT1 may be of therapeutic benefit for patients with several malignancies including lung cancer 10–13. Our findings suggested that DNMT1 may act as a potential new target that mediated the anti-lung cancer properties of β-elemene. Study demonstrated that DNA methylation was one of an early step in cancer development and increased DNMT1 expression could be considered as a critical step in the oncogenic transformation of lung epithelial cells 10. Thus, overexpression of DNMT1 results in epigenetic changes of tumour suppressor genes and ultimately results in tumourigenesis 17. Further studies are needed to elucidate the detailed mechanism by which regulation of DNMT1 controls NSCLC cell growth.

Intriguingly, our results also suggested the role of ERK1/2 and AMPKα signalling pathways in mediating the effect of β-elemene on inhibition of DNMT1 protein. Note that inactivation ERK1/2 signalling has been shown to be involved in the inhibition of DNMT1 in other studies 35,36, which was different from our findings. On the contrary, one study showed that activation of ERK1/2 by β-elemene involved in the inhibition of lung cancer cell growth 21. The discrepancy remains unclear; different stimuli, cell lines used and environment exposed may be account for this, which need to be determined in the future studies. Furthermore, our results demonstrated an additive effect in inhibition of DNMT1 expression in the presence of metformin and β-elemene. This was for the first time we observed the down-regulation of DNMT1 by metformin. There were no reports demonstrating the link of AMPK signalling and DNMT1 expression. Our findings provided the insight into the connection between AMPK signalling and this oncogenic protein expression affected by β-elemene, and also highlighted the tumour suppressor role of AMPKα that enhanced the anti-tumour effect of β-elemene. More studies are required to further elucidate the synergy of combining β-elemene with metformin in this process.

Furthermore, our results indicated the causative role of Sp1 transcriptional factor in mediating the effects of β-elemene on DNMT1 and cell growth, suggesting that Sp1 was an upstream of DNMT1. Previous data showed that DNMT1 gene promoter contained Sp1 binding sites, which regulated the expression and function of DNMT1 in several cell systems 17–19. Sp1 binds to GC-rich motifs of promoters and regulates genes involved in tumour growth, apoptosis and angiogenesis; thus, the functional status of Sp1 may affect the therapeutic response of anti-angiogenic strategies for human cancers 37. We for the first time demonstrated that β-elemene inhibited DNMT1 expression through targeting Sp1 highlighted a unique role of this transcriptional factor in this process. Nevertheless, the truly links and interactions of Sp1 and DNMT1 need to further explore.

The DNA damage response (DDR) and reading frame (ARF)/p53 pathways have been recognized as important oncogene-provoked anticancer barriers in tumourigenesis and cancer development leading to cellular senescence. Oncogenic stimulation triggers the DDR and induces the ARF signalling, both of which can activate the p53 pathway and provide intrinsic obstacles to tumour progression 38,39. In this study, our results suggested that β-elemene inhibited the NSCLC cells irrespective of p53 status. Limited data demonstrated the links of β-elemene and p53 status, one study showed that targeting human lung cancer cells by β-elemene was reported through both p53-dependent and -independent pathways 40. Thus, more in depth experiments are required to further explore the true role of p53 involving in the anti-tumour effects of β-elemene.

Collectively, our results show that β-elemene inhibits NSCLC cell growth *via* ERK1/2- and AMPKα-mediated inhibition of transcription factor Sp1, followed by reduction in DNMT1 protein expression. Overexpression of DNMT1 reverses the effect of β-elemene on growth of NSCLC cells. The reciprocal ERK1/2 and AMPKα signalling pathways contribute to the overall responses of β-elemene. In addition, blockade of Akt signalling and concomitantly inhibition of DNMT1 expression by metformin augment the effect of β-elemene (Fig.6E). This study reveals a novel mechanism by which β-elemene inhibits growth of NSCLC cells.

## Supporting Information

**Data S1** beta-elemene had no effect on phosphorylation of EGF-R in lung cacner cells. Please note that this is for reviewer only.
